# Dr. Bagadia, Sir, is No More

**DOI:** 10.4103/0973-1229.58815

**Published:** 2010

**Authors:** Ajai R. Singh

**Affiliations:** * *Editor, Mens Sana Monographs, Mens Sana Research Foundation, 14, Shiv Kripa, Trimurty Road, Nahur, Mulund (W), Mumbai-400 080, India. E-mail: mensanamonographs@yahoo.co.uk*

**Figure:**
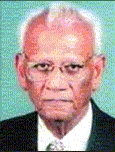
V. N. Bagadia (1922-2009)

With misty eyes, I have to report the sad demise of one at whose feet I learnt the fundamentals of clinical psychiatry, and also what it means to be a caring and compassionate human being.

Dr V. N. Bagadia [5.12.1922 – 17.8.2009] was one of the sharpest clinicians in post independence psychiatry in India. His pioneering spirit to make psychiatry recognized as an important medical discipline in the face of denial and neglect by his medical peers was well matched by his infectious zeal and enthusiasm, which could not but rub off on his students and colleagues. That icon of modern psychiatry in India, a benevolent patriarch who did so much for the branch and for all of us who have followed in his wake, and who benefit so immensely from what he bequeathed us so generously -- that giant is no more.

He headed our Honorary International Advisory Board at MSM [http://www.msmonographs.org/aboutus.asp] and was always solicitous of its welfare. In his quiet, gentle voice, he would often say, ‘ Why have you to write so difficult? Make it simple.’ Knowing the earnestness behind his advice, I felt blessed to have such counsel.

I took my first halting steps in clinical psychiatry under his care. Coming from a six months’ residency in Dr Doongaji’s unit at KEM, known for its formidable clinical case presentations and smart and clever diagnostics, I was fascinated with the humane and compassionate touch to clinical psychiatry, which was the hallmark of his unit and of the man.

## Formidable Reputation

Dr Bagadia had a formidable reputation and a rather stern exterior when you first accosted him. Part of this was because of the impression his colleagues and senior residents had created about the man, used as we all are to demonising/idolising others. However, what I found endearing about the man was his gentle, sing-song voice, almost lulling a person’s anxieties to tranquillity. He would not force his opinion on others, and yet others would be subtly forced to toe his line. God knows, he had his fair share of detractors, but even they could hardly question the sincerity and intensity of his devotion to psychiatry.

He was impassioned in clinical meetings; psychiatric, or otherwise. In psychiatric meetings, he bemoaned neglect of the psychosocial and psychodynamic; even as he welcomed the psychopharmacologic and biological [he himself headed the WHO Center for Psychopharmacology in India]. In medical meetings, he did not allow others to get away without understanding psychosomatic aspects of conditions like hypertension, diabetes, skin disorders, arthritis, asthma etc. When Dr Bagadia rose to speak at such occasions, internists trembled within, some even bristled, even as they craned their ears to listen to the master expound.

He was very fond of his residents and colleagues presenting papers. Sometimes one felt quality suffered at the hands of quantity. But I guess the reason was that, like all crusaders, he was a man in a hurry. He wanted to make his mark in numerous areas of psychiatry, and, like a true pioneer, often allowed enthusiasm to overtake acumen. The number of papers his unit presented at conferences was legion. During his hey days, rare was the conference whose front chairs were not adorned by his gracious presence. And rare was the paper, which did not undergo his critical scrutiny and comments.

## Hysteria and Fancy Diagnostics

The first thing that struck me about him as a junior resident was the way he oriented me to Hysterical conversion reaction. I came from a unit where for hysterical ‘fits’ the standard line of treatment was “admission, isolation, inj ‘R’, and study of primary and secondary gains”. And repeat process on readmissions. Coming under his care, his first question to me was, “have you found out any underlying depression, or anxiety? Are you handling that with psychotherapy and appropriate medication?” It brought about a whole paradigm shift in my perception of hysteria, something that spilled over to every other branch of clinical psychiatry. From labelling I transcended to care.

He was not really fond of fancy diagnoses, and smart terms often met with derision. He was more interested in understanding the deeper compulsions and underpinnings of psychiatric disorders, and wanted his students and colleagues to probe and remedy them, rather than get embroiled in academic exercises of fanciful diagnostic entities. Behind this propulsion was the intensity of a deeply caring human being who wanted to understand the frailties of the psyche, and was not content with just labels.

The personal interest he took in the welfare of his students, helping them to settle in practice, is well known. Any patient sent to him for second opinion was sure to be sent back to the original clinician, with a proper note about what he felt, and a firm reassurance to the patient/relative that they need not come back to him, for the referring consultant was competent enough to handle the case.

During the last few years, he was a shadow of his former self. Sometimes faltering in memory, sometimes going off on a tangent in meetings; his detractors appeared to have a field day. Even his erstwhile admirers kept silent in deference to his earlier achievements. But even there none could miss the crusading spirit, the need to champion the branch he loved, and the determination to make his viewpoint known -- a zeal because of which his medical peers could never write him off, and also had to grudgingly acknowledge the contributions of his branch -- for which we all, who follow in his wake, are beholden today.

## Misty Eyes Must Give Way to Steely Resolve

I said Dr Bagadia is no more, did I? I must take back those words. He lives on, and through his students, and through Indian psychiatry. And through the students of his students, to whom the knowledge and passion of this branch he has so benevolently bequeathed.

A giant oak has fallen. But so many hands rise to touch the giant oak, girdle their loins, and rise to the task of resurrecting the spirit of this giant oak. Misty eyes must give way to steely resolve, to take up the massive task of reaching psychiatry to the preeminent position it so richly deserves amongst the clinical sciences.

Only then will the great soul’s spirit rest in peace.

